# Forecast of pain degree of lumbar disc herniation based on back propagation neural network

**DOI:** 10.1515/biol-2022-0673

**Published:** 2023-09-08

**Authors:** Xinying Ren, Huanwen Liu, Shiji Hui, Xi Wang, Honglai Zhang

**Affiliations:** College of Medical Information Engineering, Guangzhou University of Chinese Medicine, Guangzhou, China

**Keywords:** lumbar disc herniation, magnetic resonance imaging, prediction model, imaging feature, pain score

## Abstract

To further explore the pathogenic mechanism of lumbar disc herniation (LDH) pain, this study screens important imaging features that are significantly correlated with the pain score of LDH. The features with significant correlation imaging were included into a back propagation (BP) neural network model for training, including Pfirrmann classification, Michigan State University (MSU) regional localization (MSU protrusion size classification and MSU protrusion location classification), sagittal diameter index, sagittal diameter/transverse diameter index, transverse diameter index, and AN angle (angle between nerve root and protrusion). The BP neural network training model results showed that the specificity was 95 ± 2%, sensitivity was 91 ± 2%, and accuracy was 91 ± 2% of the model. The results show that the degree of intraspinal occupation of the intervertebral disc herniation and the degree of intervertebral disc degeneration are related to LDH pain. The innovation of this study is that the BP neural network model constructed in this study shows good performance in the accuracy experiment and receiver operating characteristic experiment, which completes the prediction task of lumbar Magnetic Resonance Imaging features for the pain degree of LDH for the first time, and provides a basis for subsequent clinical diagnosis.

## 1 Introduction

Lumbar disc herniation (LDH) is a disease that occurs when the lumbar discs undergo degenerative changes, causing the annulus to break. As a result, the nucleus pulposus protrudes or extrudes through the posterior longitudinal ligament, either below or by passing through it, into the spinal canal. This compression of surrounding tissue can lead to some major symptoms, including low back pain (LBP), radiative pain in the legs, and numbness. The most commonly affected areas are the L4-L5 and L5-S1 discs, which account for approximately 90% of all LDH cases. The disease mainly affects people between the ages of 20 and 40, with a higher incidence in male, with a male-to-female ratio of about (10–15):1.

LBP is the predominant clinical symptom of LDH, frequently causing movement restriction in individuals aged below 45. This is the second leading cause of seeing a doctor, the fifth leading cause of hospitalization, and the third leading cause of operation [1]. The prevalence of chronic LBP among individuals aged 24–39 is 4.2%, while among individuals aged 20–59, it is 19.6% [2]. In 2015, the global prevalence of LBP among adults reached 7.3% [3]. LBP has become a major cause of disability worldwide [4,5], with approximately 1% of Americans experiencing long-term disability due to LBP, and an additional 1% experiencing temporary disability [1].

Intervertebral disc degeneration (IDD) increases with age, with over 80% of individuals aged 50 and above exhibiting degenerative changes in their intervertebral discs. Lumbar disc degeneration is widely recognized as a contributing factor to LBP. However, the pathophysiological mechanisms of discogenic LBP are not yet fully understood. Chemical and mechanical compression of nerve root inflammation is thought to be the source of neuropathic pain [6].

In the 1970s, the emergence of Magnetic Resonance Imaging (MRI) and Computed Tomography in the field of medical imaging provided advantageous techniques for diagnosing spinal disorders. Even today, MRI remains the most recommended and suitable imaging modality for diagnosing nerve root-related LDH [7]. Numerous researchers have utilized imaging techniques to reconstruct two-dimensional or three-dimensional images of the lumbar spine in LDH patients, conducting studies related to investigating the mechanisms of LDH development, evaluating treatment effectiveness, and guiding therapy through the analysis of imaging features. Their research methods encompass clinical statistical analysis as well as deep learning and machine learning techniques [8,9,10]. However, there is significant variation in the research findings, with some studies indicating a significant positive correlation between the severity of LDH and lower limb pain scores [11], while others have found that certain imaging features are unrelated to disc degeneration or pain [12].

Previous studies on the relationship between imaging features and symptoms of degenerative spinal diseases have predominantly used traditional statistical methods. Sasaki et al. employed multivariate logistic regression analysis to evaluate the association between paraspinal muscle fat infiltration and the incidence of LBP [13]. Dunsmuir et al. employed Pearson correlation analysis to investigate the direct correlation between patients’ pain scores, disability scores, and the size of disc herniation [14]. Ranger et al. utilized t-tests and Chi-square tests to assess the morphological characteristics of paraspinal muscles in individuals with unilateral LDH, by comparing the differences in paraspinal muscle morphology between the back pain group (VAS score >4) and the control group (VAS score ≤4) [15].

In recent years, owing to the ongoing advancements in artificial intelligence, researchers have also begun to explore the integration of machine learning with degenerative spinal diseases. Compared to traditional statistical methods, machine learning offers the advantage of higher model accuracy, especially when dealing with variables that exhibit complex interactions [16]. Eriksson et al. predicted pain scores in patients with LBP using a logistic regression model based on image features. The sensitivity/specificity values were 0.90/0.36, 0.88/0.4, and 0.93/0.33, with Area Under the Curve (AUC) values of 0.69, 0.75, and 0.73, respectively [17]. Su et al. developed a multi-task classification network based on ResNet50 for the automatic assessment and grading of LDH, central canal stenosis, and compressed lumbar nerve roots in lumbar axial MRI. The overall accuracies for the three tasks were 84.17% (74.16%), 86.99% (79.65%), and 81.21% (74.16%), respectively [18]. Abdollah et al. extracted gray-level co-occurrence matrix features from MRI images in the intervertebral disc and upper and lower endplate regions of 14 patients with chronic LBP and 14 healthy volunteers. They established a classification model using the random forest algorithm to distinguish between the pain group and the pain-free group [19]. Hopkins et al. analyzed cervical spine magnetic resonance images of 14 patients with myelopathic cervical spondylosis and 14 control subjects using machine learning, by training an artificial neural network model to predict the modified Japanese Orthopaedic Association scores for myelopathic cervical spondylosis [20]. Currently, artificial intelligence has been widely applied to symptom prediction in degenerative spinal diseases. However, we found that many of these studies relied on imaging omics features as inputs for the models. Nonetheless, imaging omics features cannot fully explain underlying biological mechanisms, and the specific clinical and pathological changes they correspond to are not yet known. This limitation will significantly impede subsequent research on the pathological mechanisms.

The aim of this study is to further explore the pathogenesis of pain in patients with LDH. It analyzes the imaging features of the surrounding tissues of the lumbar intervertebral disc and their correlation with the Oswestry disability index (ODI) scores in LDH patients. Significant correlated structural features will be selected to train a back propagation (BP) neural network model. The model will utilize the MRI imaging features of the lumbar spine to predict the degree of pain in LDH, and identify important imaging biomarkers for the assessment of LDH symptoms and guidance of treatment.

This study is organized as follows: Section 2 provides detailed information on the case sources, inclusion and exclusion criteria, collection of imaging features, collection of questionnaires, statistical analysis methods, and construction methods of the BP neural network. Section 3 presents the statistical analysis results of the basic characteristics of the cases, the statistical analysis results of imaging characteristics, the correlation analysis results between imaging characteristics and symptom scores, as well as the results of the BP neural network model. Section 4 primarily focuses on the analysis and discussion of imaging features that are strongly correlated with symptom scores, as well as the analysis and discussion of the experimental results of the neural network.

## 2 Materials and method

### 2.1 Research method

The aim of this study is to describe and analyze the demographic and imaging features of LDH. The goal is to statistically analyze the correlation between imaging features of the surrounding tissues of the lumbar disc and the severity of pain. Significant correlated structural features will be selected and used to train a BP neural network model to predict the degree of pain in LDH using lumbar spine MRI imaging features. The aim of this study is to identify important imaging biomarkers for assessing LDH symptoms and guiding treatment. The research method chosen is a case series analysis, which involves the description, analysis, and summary of a series of demographic, clinical, and epidemiological features of a group of individuals with the same disease. This analysis method is used to analyze the clinical manifestations of a specific disease, evaluate the effectiveness of prevention and treatment measures, and contribute to the accumulation of clinical information, summarization of clinical experience, and improvement of disease diagnosis and technological research.

### 2.2 Study subjects

#### 2.2.1 Source of study subjects

Ninety cases of LDH patients who underwent lumbar spine MRI examinations at Guangdong Provincial Hospital of Traditional Chinese Medicine from February 2022 to April 2022 were collected as study subjects. The hospital had a total outpatient volume of 7.22 million visits in 2021, ranking second in China in terms of outpatient volume. LDH accounted for 25–40% of orthopedic inpatients with lower back and leg pain. The imaging department was equipped with GE Signa 3.0T and Siemens Verio 3.0T superconductive MRI machines, a dedicated medical imaging and image processing laboratory, a picture archiving and communication system (PACS) for medical image storage and communication, and high-performance image workstations capable of offline processing and comprehensive analysis software. All patients underwent lumbar spine MRI scans using Siemens Verio 3.0T superconductive MRI. The lumbar spine MRI images were stored in the form of digital imaging and communications in medicine files.


**Informed consent:** Informed consent has been obtained from all individuals included in this study.
**Ethical approval:** The research related to human use has been complied with all the relevant national regulations, institutional policies and in accordance with the tenets of the Helsinki Declaration, and has been approved by the Ethics Committee of Guangdong Provincial Hospital of Traditional Chinese Medicine (approval number: YE2022-037-01).

#### 2.2.2 Inclusion criteria

(1) Age of 18–80 years old;
(2) All patients were diagnosed as LDH by medical history, physical examination, and imaging examination;
(3) Patients who had lumbar MRI in the past (within 1 month) were checked by PACS system of Guangdong Provincial Hospital of Traditional Chinese Medicine;
(4) Informed consent was obtained from the patients.

#### 2.2.3 Exclusion criteria

(1) Those who had a history of lumbar surgery, which affected the judgment of the results;
(2) Patients with lumbar compression fracture;
(3) LDH caused by acute violent injury;
(4) Combined with other spinal diseases (including lumbar anterior spondylolisthesis, tumor, tuberculosis, infection, scoliosis or kyphosis, metabolic bone disease, etc.).

### 2.3 Collection of basic information

By accessing the medical record system of Guangdong Provincial Hospital of Traditional Chinese Medicine, basic information of LDH patients was collected, including disease name, hospitalization number/outpatient number, gender, age, height, weight, duration of illness, chief complaint, present medical history, past medical history, and Western medicine diagnostic basis.

### 2.4 Acquisition of imaging features

All patients underwent lumbar MRI scans with a Magnetom Trio 3.0T MRI scanner (Siemens, Germany). All patients were in the supine position. Scan parameters were pulse repetition time of 575 ms, echo time of 18.5 ms, field of view of 160 mm × 160 mm, voxel size of 0.4 mm × 0.4 mm × 4.0 mm, and layer spacing of 0.3 mm.

The imaging data were exported from the hospital’s PACS system, and the patients’ lumbar MRI images (including sagittal T1, T2 sequences, and transverse T2 scans) were collected. The imaging features data were collected by observing the image pictures to highlight the most severe segments and the ITK-SNAP software [21] was used to measure the highlighted segments in the image pictures.

#### 2.4.1 Pfirrmann classification

**Figure 1:**
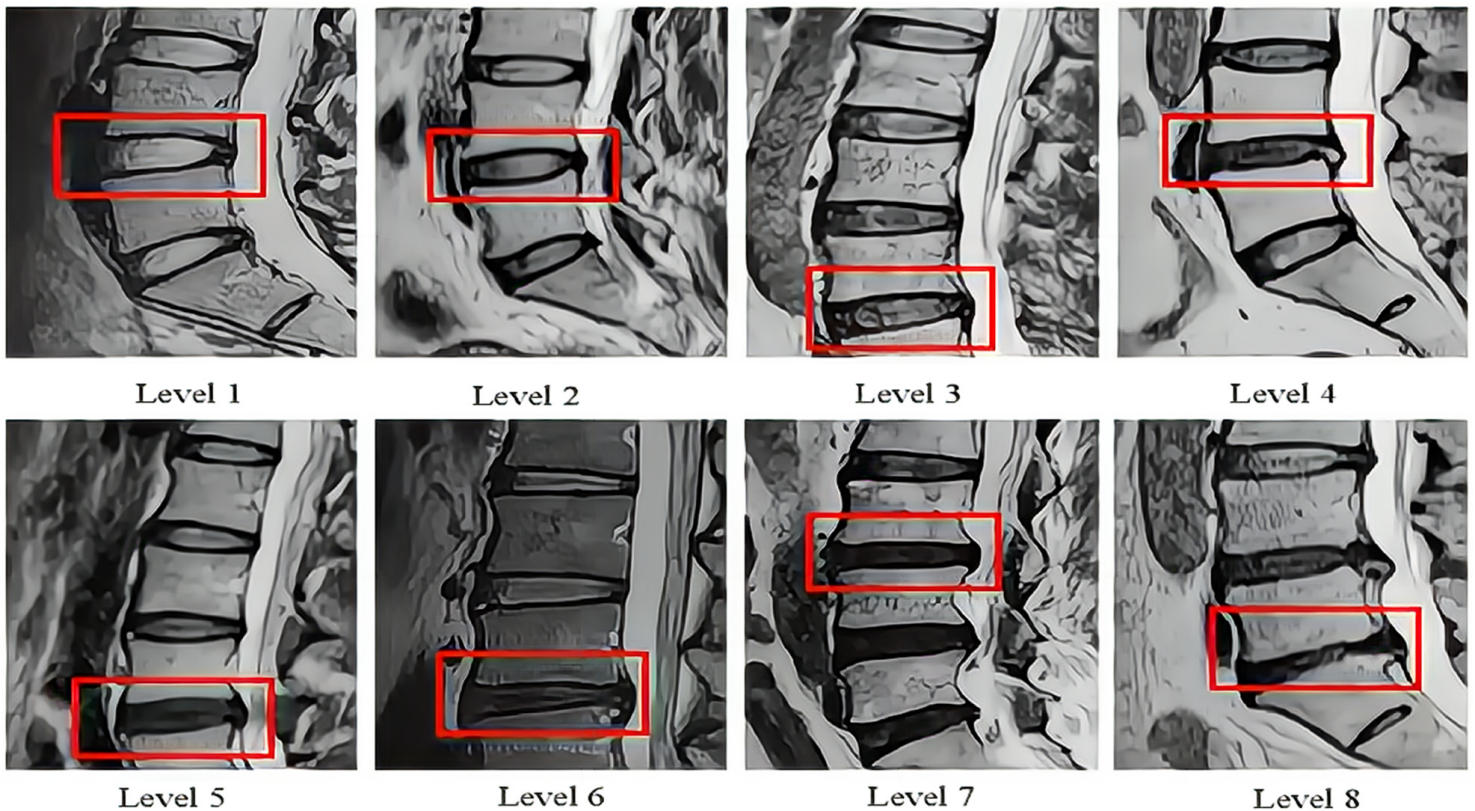
Pfirrmann classification.

Pfirrmann classification is the grade describing the degeneration of lumbar intervertebral disc, and the higher the grade, the more serious the degeneration. In this study, the modified Pfirrmann classification was selected, with a total of 8 grades [22,23] (Figure 1).

The position of the red frame is the diseased intervertebral disc. The degree of lumbar disc degeneration is described using the modified Pfirrmann grading system, which consists of 8 levels.

Level 1: The signal of nucleus pulposus and medial annulus fibrosus was uniform high signal, which was significantly different from that of cerebrospinal fluid and posterior annulus fibrosus, and the height of intervertebral disc was normal.

Level 2: The signal of nucleus pulposus and medial annulus fibrosus was high (stronger than presacral fat, less than cerebrospinal fluid) or there was horizontal fissure in nucleus pulposus. The signal difference between the inner and outer fibers of the posterior ring was obvious, and the height of the intervertebral disc was normal.

Level 3: The signals of the nucleus pulposus and the medial annulus fibrosus were high (lower than that of the anterior sacral fat), and the signals of the fibers inside and outside the posterior annulus were different, and the intervertebral disc height was normal.

Level 4: The signal of nucleus pulposus and medial annulus fibrosus was moderately high (slightly stronger than that of lateral annulus fibrosus), the difference of posterior annulus fibrosus signal was not obvious, and the height of intervertebral disc was normal.

Level 5: The signal of nucleus pulposus and medial annulus fibrosus was low and high signal (equal to lateral annulus fibrosus), and the signal difference between the medial and lateral fibers of the posterior annulus fibrosus was not obvious, and the height of intervertebral disc was normal.

Level 6: The signal of nucleus pulposus and medial annulus fibrosus was low signal, and the signal difference between the medial and lateral fibers of the posterior annulus fibrosus was not obvious, and the height of the intervertebral disc was reduced by <30%;

Level 7: The signal of nucleus pulposus and medial annulus fibrosus was low signal, and the signal difference between the medial and lateral fibers of the posterior annulus fibrosus was not obvious. The height of the intervertebral disc was reduced by 30%–60%.

Level 8: The signal of nucleus pulposus and medial annulus fibrosus was low signal, and the signal difference between the medial and lateral fibers of the posterior annulus fibrosus was not obvious, and the height of the intervertebral disc was reduced by >60%.

#### 2.4.2 Modic classification

**Figure 2:**
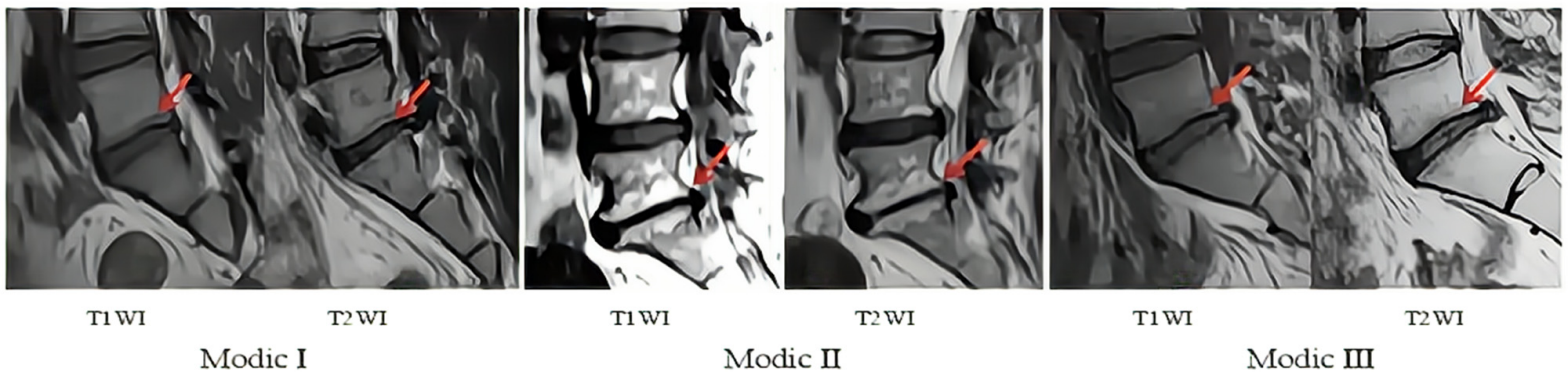
Modic classification.

Modic classification [24,25,26] (Figure 2) describes the degree of vertebral endplate degeneration.

The position of the red frame is the diseased endplate. Type I changes (T1 low signal, T2 high signal) correspond to vertebral marrow edema. Type II changes (T1 high signal, T2 high signal or iso-signal) represent fatty replacement of the marrow. Type III changes (T1 low signal, T2 low signal) are observed in vertebral osteosclerosis.

#### 2.4.3 Michigan State University (MSU) regional localization

**Figure 3:**
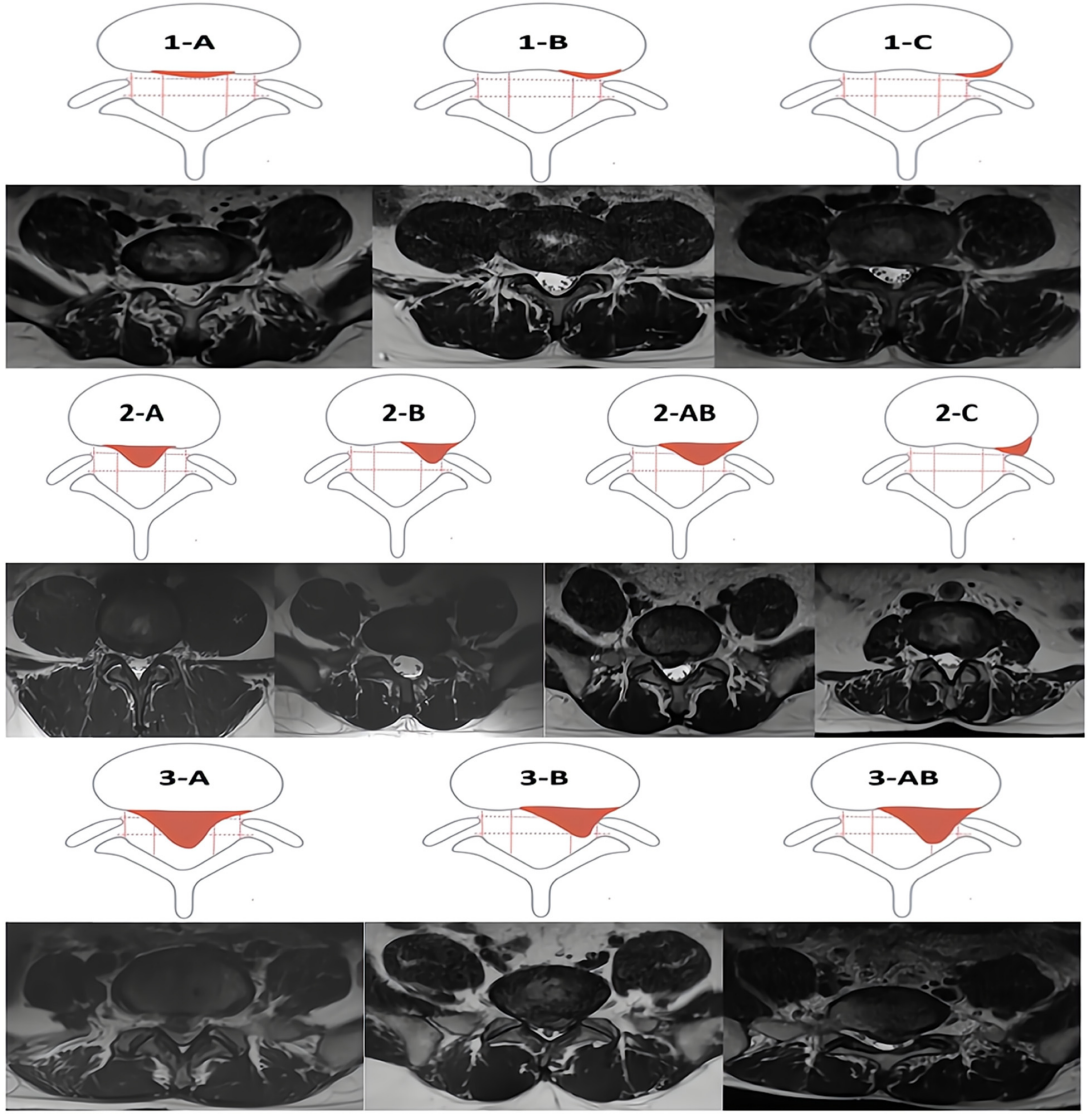
MSU regional localization.

The upper column is a schematic of the measurement method, and the lower column is the corresponding T2W TSE image. The anatomical level with the most prominent nucleus pulposus was selected from the T2 sequence. It is the evaluation criteria for the size and location of lumbar disc protrusions inside and outside the spinal canal. The specific location and degree of protrusion of the herniated nucleus pulposus within the spinal canal are defined by different region grades, with a total of ten region grades. The anatomical plane with the most prominent herniated nucleus pulposus is selected from the T2 sequence. Two lines are drawn on both sides of the superior and inferior articular processes, and if the protrusion does not exceed the line connecting the superior articular processes, it is classified as grade 1. If it does not exceed the line connecting the inferior articular processes but exceeds the line connecting the superior articular processes, it is classified as grade 2. If it exceeds the line connecting the inferior articular processes, it is classified as grade 3. According to the location of the protrusion, it is divided into four regions: central region (A region), adjacent region (AB region), outer region (B region), and far outer region (C region) [27] (Figure 3).

#### 2.4.4 Sagittal diameter index (SI)

**Figure 4:**
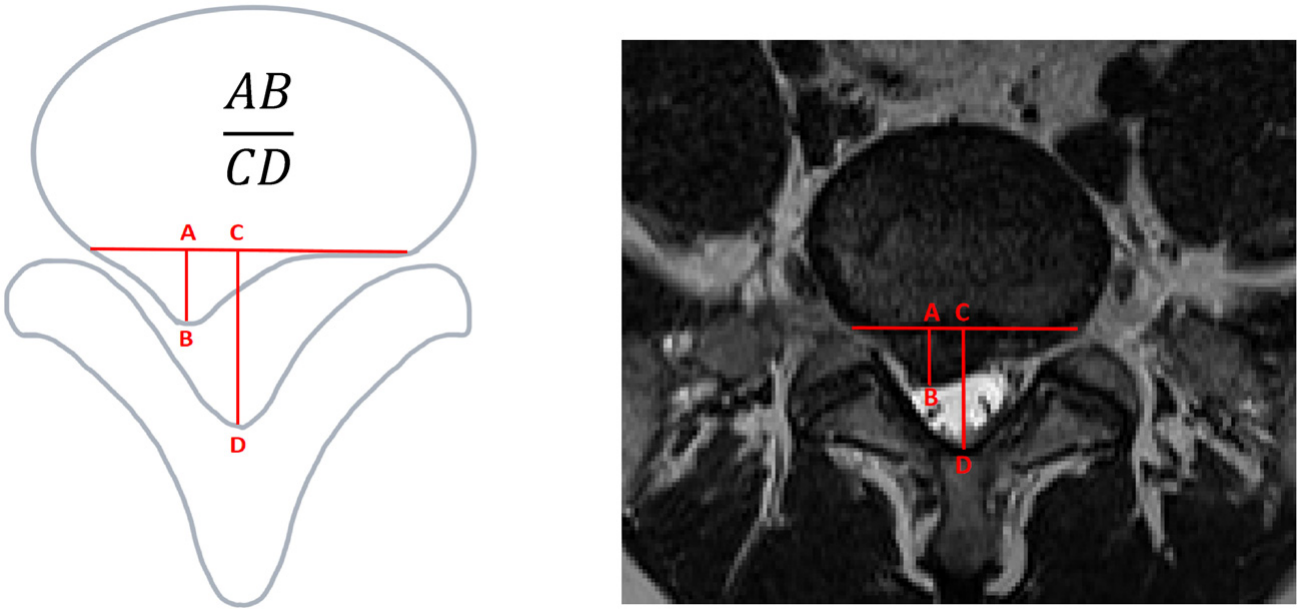
Sagittal diameter index (SI).

SI [28] (Figure 4) is calculated as the ratio of the maximum sagittal diameter (AB) of the disc protrusion to the maximum sagittal diameter (CD) of the spinal canal. It provides a measurement of the position of the protrusion apex within the spinal canal. A higher SI ratio indicates a more severe disc protrusion that occupies a larger portion of the spinal canal.

#### 2.4.5 Sagittal diameter/transverse diameter index (STI) [28] (Figure 5)

**Figure 5:**
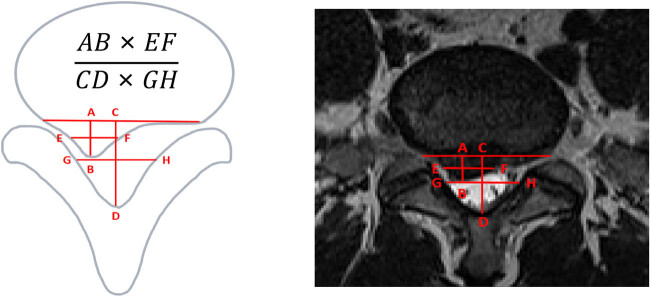
Sagittal diameter/transverse diameter index.

STI [28] (Figure 5) is calculated as the ratio of the product of the maximum sagittal diameter (AB) and transverse diameter (EF) of the disc protrusion to the product of the maximum sagittal diameter (CD) and transverse diameter (GH) of the spinal canal. It provides a measurement of the extent to which the disc protrusion occupies the spinal canal. A higher STI ratio indicates a more severe disc protrusion with greater occupation of the spinal canal.

#### 2.4.6 Transverse diameter index (TI)

**Figure 6:**
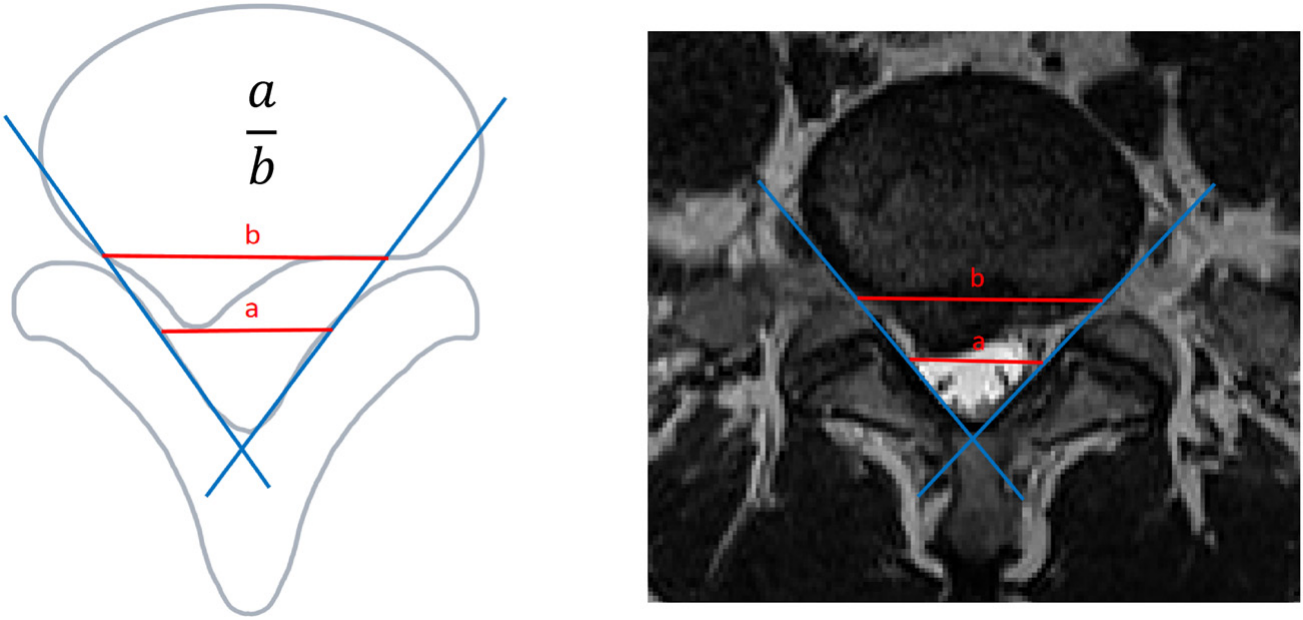
Transverse diameter index.

TI is calculated as the ratio between the length of a parallel line (a) at the apex of the disc protrusion and the length of the posterior line (b) of the corresponding vertebra (Figure 6). This index provides a measure of the extent to which the disc protrusion occupies the spinal canal. A smaller TI ratio indicates a more severe disc protrusion with greater occupation of the spinal canal.

#### 2.4.7 Angle between nerve root and protrusion

**Figure 7:**
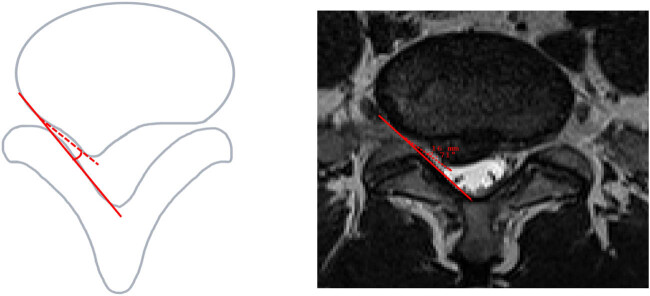
Angle between nerve root and protrusion.

The AN angle is determined by the connection between the lateral edge of the herniated nucleus pulposus and the lateral edge of the vertebra, along with the extension line of the inner wall of the spinal canal [29,30] (Figure 7). A smaller angle correlates with a greater degree of nerve root compression, indicating more severe compression.

#### 2.4.8 Thickness of ligamentum flavum

**Figure 8:**
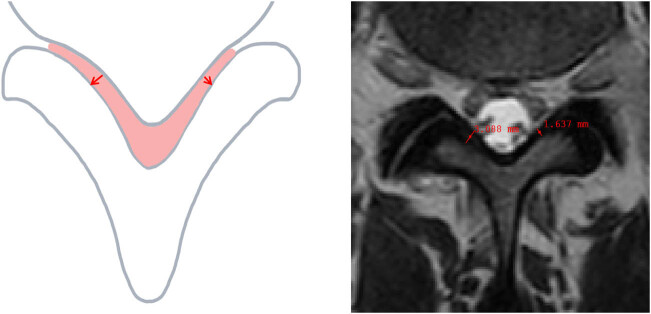
Thickness of ligamentum flavum.

The ligamentum flavum is a ligament that connects the superior lamina to the superior and lateral edges of the inferior lamina, originating from the inferior and medial edges of the superior lamina [31] (Figure 8). In cases where the measurements of bilateral ligamentum flavum differ, the larger value is chosen as the measurement result.

### 2.5 ODI score collection

For eligible patients who met the research criteria, the research plan, objectives, significance, etc., were explained in detail, and their consent was obtained. After obtaining their willingness to participate, an online questionnaire (using the Wenjuanxing platform, https://www.wjx.cn) was used for questionnaire collection. The link to the online questionnaire, which combined the informed consent form and the ODI scoring form, was sent to the patients. Patients were required to read the informed consent form, provide an electronic signature, and click “Agree” to proceed to the formal answering interface. For older patients who were not proficient in operating the system, family members could assist them in completing the questionnaire.

The ODI scoring form [32] is used to assess the severity of pain in patients. It consists of 10 items and is rated on a 6-point scale, with scores ranging from 0 to 5. The scores for each item are summed to obtain a total score, calculated as (score obtained/5 × number of questions answered) × 100%. Scores below 15 indicate mild pain, scores between 15 and 30 indicate moderate pain, and scores above 30 indicate severe pain.

### 2.6 Statistical analysis

This study used SPSS Statistics 25.0 for data statistical analysis. The normality of quantitative variables was assessed using the Shapiro–Wilk test. If the data adheres to a normal distribution, it is presented as \[\bar{x}]\] ± *s* standard deviation. Pearson correlation analysis was employed for examining correlations. In cases where the data satisfy the assumption of homogeneity of variance, one-way analysis of variance was conducted on multiple independent samples, followed by paired comparisons using the Bonferroni method. Conversely, if the data do not conform to a normal distribution, it is presented as the median (quartile range) [M(P25, P75)], and subsequent correlation analysis is carried out using the Spearman rank correlation analysis. When the assumption of homogeneity of variance is violated, the Kruskal–Wallis *H* test is employed to compare multiple independent samples, followed by pairwise comparisons using Bonferroni correction. The adjusted significance level for all tests was set at *P* < 0.05. For qualitative variables deemed statistically significant, the count (percentage; %) was used for representation. Group comparisons were performed using the chi-square test or Fisher’s exact test, with Bonferroni correction applied for pairwise comparisons. The significance level of pairwise comparisons was adjusted using the α distribution method. Correlation analysis was utilized to examine the relationship between statistically significant image features identified from inter-group comparisons. Subsequently, various artificial neural network models were trained and employed for prediction using these noteworthy features.

### 2.7 Pain degree prediction model of LDH based on BP neural network model

**Figure 9:**
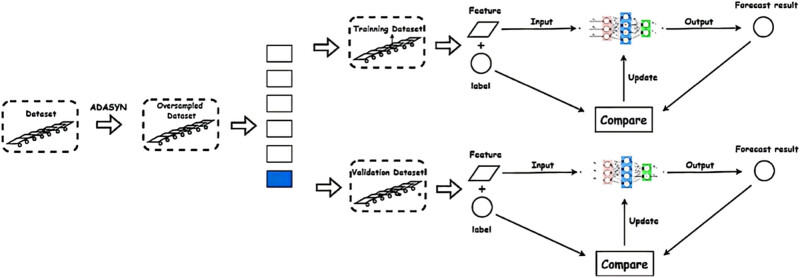
Neural network training.

The BP neural network model learns certain patterns from existing data and is used to predict subsequent classification tasks. Specifically, we perform ADASYN oversampling on the existing data to obtain augmented data. Then, in each round, we repeatedly train our neural network model through *k*-fold cross-validation (Figure 9).

#### 2.7.1 Data pre-processing

After statistical analysis, Pfirrmann classification, MSU protrusion size grading, MSU protrusion location classification, SI, STI, TI, and AN angle were selected as the data parameters, and these data were subjected to certain processing. All data were individually standardized using the z-score normalization formula.

(1) \[{X}_{* }=\frac{x-\mu }{\delta }.]\]

#### 2.7.2 Data oversampling

Due to the limited sample size of only 90 cases, there is a need to employ oversampling techniques to increase the sample size and balance the frequency of different categories. Oversampling techniques typically involve two approaches. The first approach is random sampling, which involves randomly sampling within the data space. The second approach involves data synthesis, where new data are generated based on the relationships within the existing data. In this model, the ADASYN oversampling algorithm is used. ADASYN is an adaptive synthetic oversampling method and represents one approach to data synthesis.

#### 2.7.3 *k*-fold cross validation

**Figure 10:**
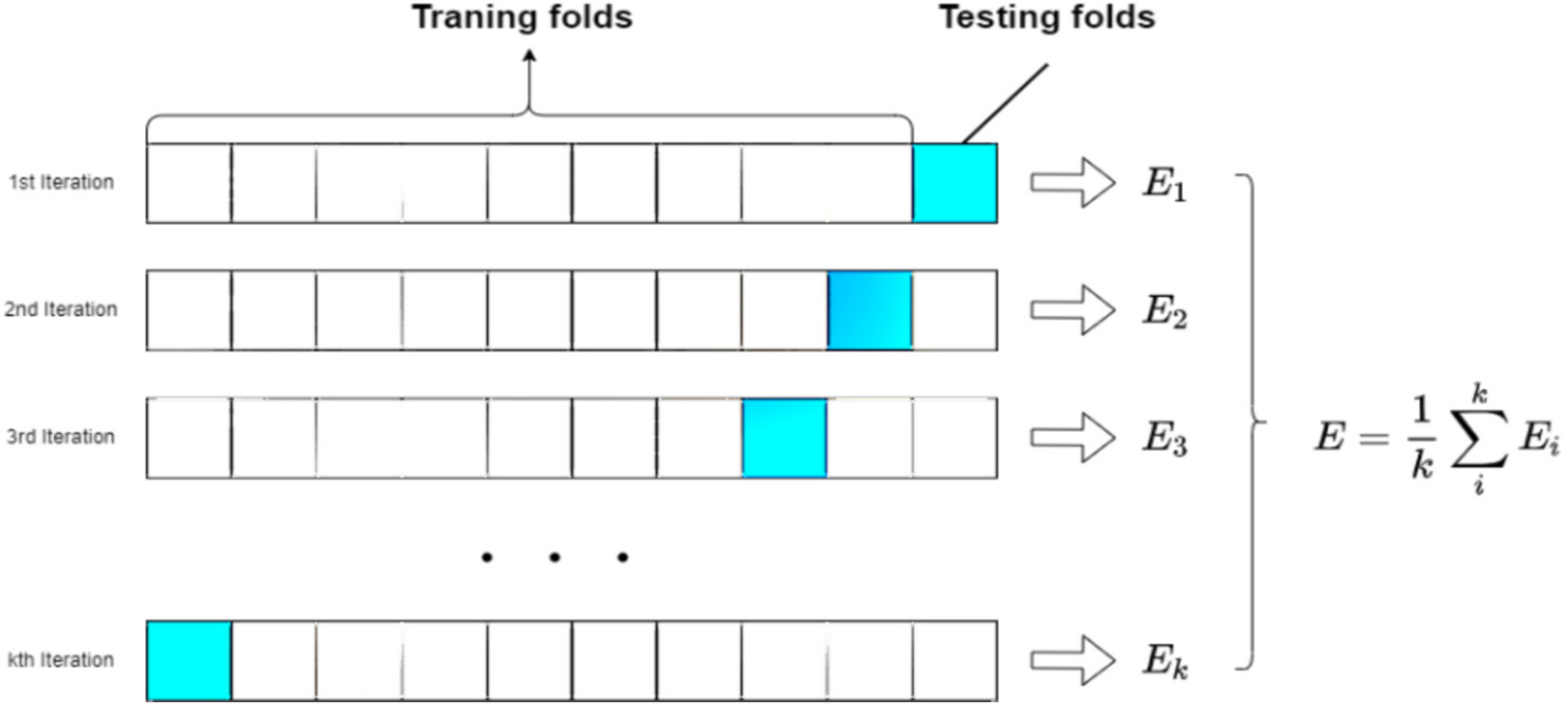
Schematic representation of *k*-fold cross validation.

To tackle the challenge of limited data size, the model adopts the *k*-fold cross-validation method to train the artificial neural network (Figure 10). The value of *k* is set to 10. In each iteration, we divide all samples into 9/10 (81 cases) for training the neural network, while the remaining 1/10 (9 cases) is used as the test set.

#### 2.7.4 BP neural network construction

**Figure 11:**
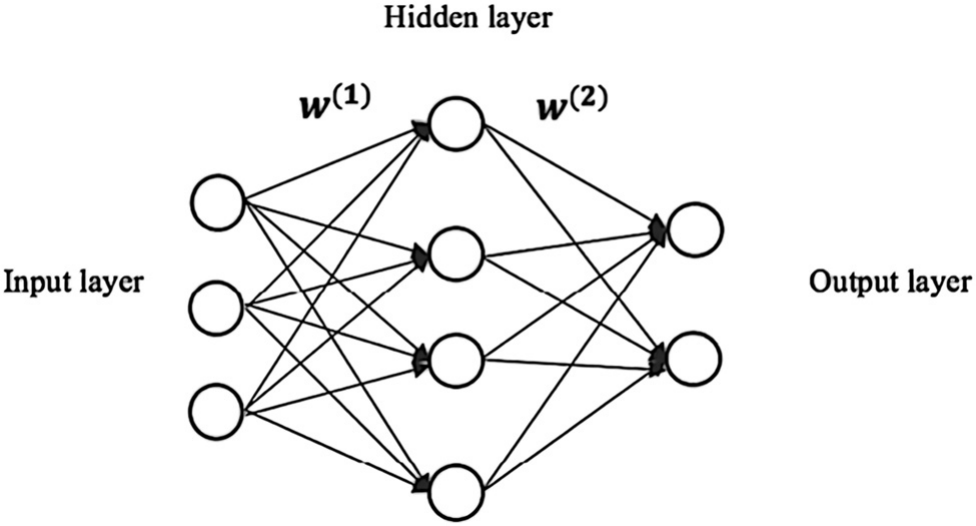
Schematic representation of a single hidden layer artificial neural network.

The single hidden layer BP neural network is used (Figure 11). Each layer represents a collection of nodes, where the input layer represents the features and the output layer represents the target variable. In multi-classification tasks, the output layer has the same number of nodes as the predicted categories, and the value assigned to each node represents the corresponding class probability. The adjacent layers are connected by a parameterized edge, which transmits information. The parameters are denoted as the first dimension, which denotes the number of neurons in the previous layer, while the second dimension indicates the number of neurons in the next layer.

The input layer consists of seven neurons: Pfirrmann classification, MSU regional positioning (MSU protrusion size classification and MSU protrusion location classification), SI, STI, TI, AN angle, the hidden layer contains 16 neurons, and the output layer consists of 3 neurons (mild group, moderate group, and severe group) for classification output.

The activation function chosen is the Rectified Linear Unit (ReLU) activation function. The ReLU sets the output of some neurons to zero, resulting in network sparsity. It also reduces the interdependence of parameters, alleviating the occurrence of overfitting problems.

(2) \[\text{ReLu}\left(x)\text{}\left=\text{}\max (0\left,x).]\]

In the output layer, the outputs are passed through the softmax function to obtain the confidence scores for each class, and the class with the highest confidence score is considered as the classification result.

(3) \[\text{Softmax}({z}_{i})=\frac{{e}^{{z}_{i}}}{{\sum }_{c=1}^{C}{e}^{{z}_{c}}}.]\]

The initial neural network is not stable, and its prediction efficiency is low. To improve the prediction accuracy of the neural network, we use the crossentropy loss function.

(4) \[\text{Loss}=-\frac{1}{N}\mathop{\sum }\limits_{i=0}^{N-1}\mathop{\sum }\limits_{k=0}^{K-1}{y}_{i,k}\mathrm{ln}{p}_{i,k}.]\]

## 3 Results

This study included a total of 90 patients with LDH, including 46 males (51.1%) and 44 females (48.9%). The distribution of the herniated discs was as follows: L2/3: 1 case (1%), L3/4: 2 cases (2%), L4/5: 31 cases (34.4%), and L5/S1: 56 cases (62.2%). Based on the ODI, the patients were divided into the following groups: mild group with 40 cases (44.4%); moderate group with 29 cases (32.2%); and severe group with 21 cases (23.3%). A comprehensive statistical analysis was conducted on the gender, age, height, weight, and duration of illness among the three groups of enrolled cases (Table 1), and no statistically significant differences were observed (*P* > 0.05), allowing for intergroup comparisons.

**Table 1:** Gender, age, height, weight, and course of disease were compared among the groups

	Gender		Age (years)	Height (cm)	Weight (kg)	Course of disease (month)
	Male	Female				
Mild group	16 (40.00)	24 (60.00)	39.65 ± 11.84	164.94 ± 8.68	61.00 (50.50, 70.00)	6.00 (3.25, 35.50)
Moderate group	17 (58.60)	12 (41.40)	37.34 ± 11.28	166.83 ± 8.16	65.00 (57.00, 71.00)	7.00 (3.00, 36.00)
Severe group	13 (61.90)	8 (38.10)	40.05 ± 11.84	166.81 ± 10.61	70.00 (55.00, 74.50)	7.00 (3.00, 42.00)
*X²/F/H*	3.610		0.439	0.486	2.074	0.638
*P*	0.164		0.646	0.617	0.355	0.727

Gender distribution among the groups was analyzed using frequency counts, represented as (number [%]) and tested with a chi-square test (*X* ^2^ = 3.610, *P* = 0.164), showing no statistically significant differences. Shapiro–Wilk tests were conducted to assess normal distribution and tests for homogeneity of variances among the groups.

Among the mild group, moderate group, and severe group, there were significant difference in the overall mean value of Modic classification, Pfirrmann classification, MSU protrusion size classification, MSU protrusion location classification, SI, STI, TI, and AN angle. But there was no significant difference in ligamentum flavum thickness among the three groups (*F* = 1.499, *P* = 0.229 > 0.05), which was excluded as shown in Table 2.

**Table 2:** Overall statistical analysis of imaging features

		Mild group	Moderate group	Severe group	*F/H/X²*	*P*
Modic classification	None	34 (85.0)	18 (62.1)	8 (38.1)	18.631	0.001
	Ⅰ	2 (5.0)	1 (3.4)	1 (4.8)		
	Ⅱ	3 (7.5)	10 (34.5)	11 (52.4)		
	Ⅲ	1 (2.5)	0 (0)	1 (4.8)		
Pfirrmann classification	1	2 (5.0)	0 (0)	0 (0)	56.868	0.000
	2	2 (5.0)	0 (0)	0 (0)		
	3	7 (17.5)	2 (6.9)	0 (0)		
	4	15 (37.5)	7 (24.1)	0 (0)		
	5	11 (27.5)	8 (27.6)	6 (28.6)		
	6	3 (7.5)	12 (41.4)	2 (9.5)		
	7	0 (0)	0 (0)	9 (42.9)		
	8	0 (0)	0 (0)	4 (19.0)		
MSU protrusion size classification	1	37 (92.5)	17 (58.6)	3 (14.3)	43.454	0.000
	2	3 (7.5)	12 (41.4)	12 (57.1)		
	3	0 (0)	0 (0)	6 (28.6)		
MSU protrusion location classification	1a	32 (80.0)	12 (41.4)	1 (4.8)	55.406	0.000
	1b	4 (10.0)	5 (17.2)	2 (9.5)		
	1c	1 (2.5)	6 (20.7)	0 (0)		
	2a	3 (7.5)	5 (17.2)	3 (14.3)		
	2b	0 (0)	1 (3.4)	3 (14.3)		
	2ab	0 (0)	0 (0)	4 (19.0)		
	2c	0 (0)	0 (0)	2 (9.5)		
	3a	0 (0)	0 (0)	2 (9.5)		
	3b	0 (0)	0 (0)	1 (4.8)		
	3ab	0 (0)	0 (0)	3 (14.3)		
Thickness of ligamentum flavum (cm)		0.31 ± 0.09	0.32 ± 0.10	0.35 ± 0.11	1.499	0.229
SI		0.23 ± 0.05	0.36 ± 0.04	0.53 ± 0.06	263.190	0.000
STI		0.15 ± 0.05	0.25 ± 0.06	0.42 ± 0.12	61.056	0.000
TI		0.78 ± 0.05	0.66 ± 0.05	0.48 ± 0.07	195.220	0.000
AN angle (°)		26.33 ± 4.27	14.87 ± 5.59	4.20 ± 3.86	162.481	0.000

The frequency statistics of Modic classification, Pfirrmann classification, MSU protrusion size classification, and MSU protrusion location classification between groups were expressed as (number [%]). Chi-square test was used.

The Modic classification, Pfirrmann classification, MSU protrusion size classification, and MSU protrusion location classification are subsequently pairwise multiple comparisons, which were conducted using the α division method. The SI, STI, TI, and AN angle underwent pairwise multiple comparisons utilizing the Bonferroni method. The results showed that there were statistically significant differences (adjusted *P* < 0.05) in pairwise comparisons of the Pfirrmann classification, MSU protrusion size classification, MSU protrusion location classification, SI, STI, TI, and AN angle between the mild, moderate, and severe groups. These variables were included in the correlation analysis. However, there was no statistically significant difference (adjusted *P* = 0.243 > 0.05) in the Modic classification between the moderate and severe groups, and it was excluded. Please refer to Table 3 for details.

**Table 3:** Multiple comparison of imaging features and statistical analysis of *P* value

	Mild group vs moderate group	Mild group vs severe group	Moderate group vs severe group
Modic classification	0.015	0.000	0.243
Pfirrmann classification	0.011	0.000	0.000
MSU protrusion size classification	0.001	0.000	0.000
MSU protrusion location classification	0.002	0.000	0.002
SI	0.000	0.000	0.000
STI	0.000	0.000	0.004
TI	0.000	0.000	0.000
AN angle (°)	0.000	0.000	0.000

**Figure 12:**
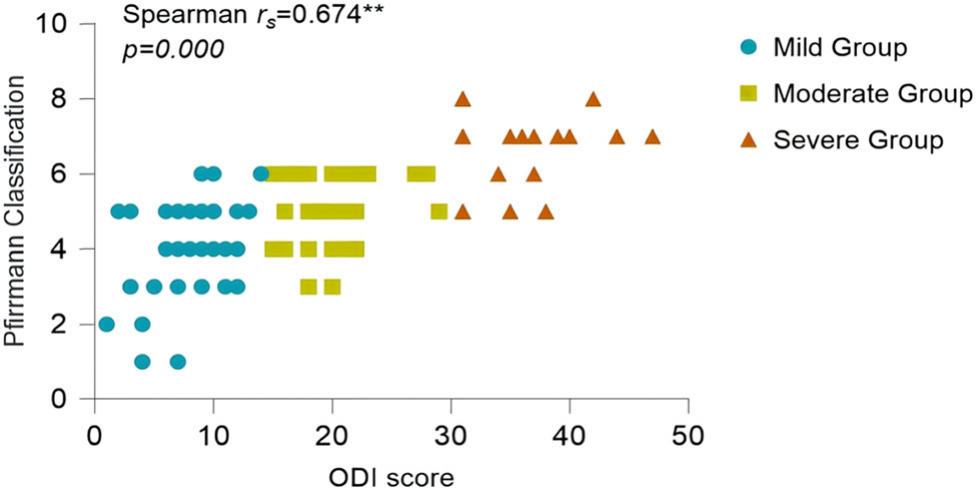
Correlation analysis between Pfirrmann classification and ODI.

**Figure 13:**
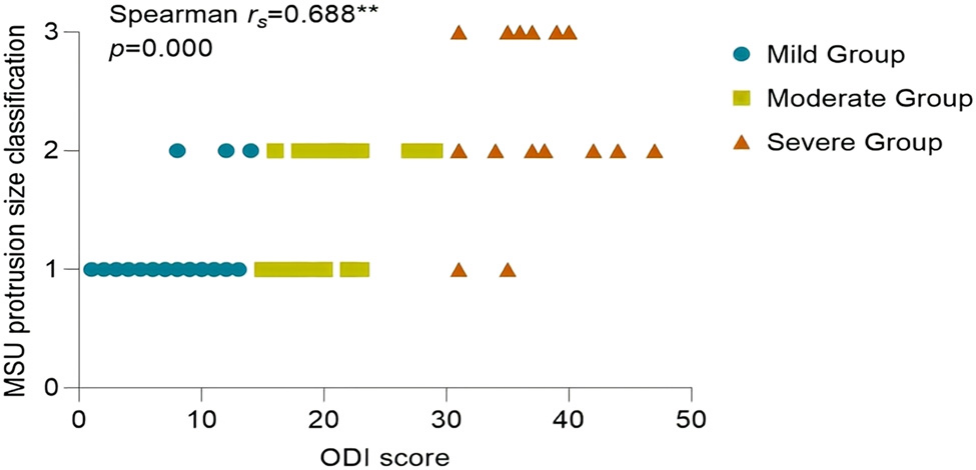
Correlation analysis between MSU protrusion size classification and ODI.

**Figure 14:**
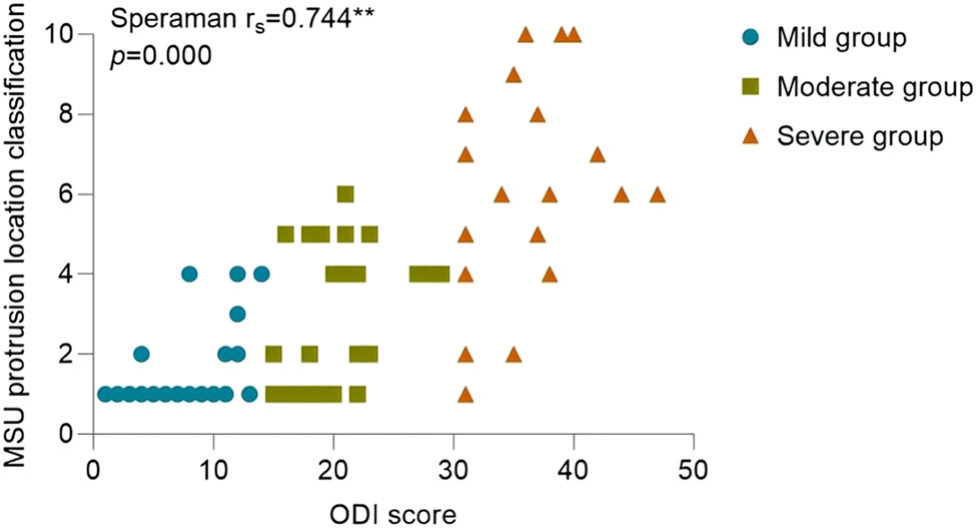
Correlation analysis between MSU protrusion location classification and ODI.

**Figure 15:**
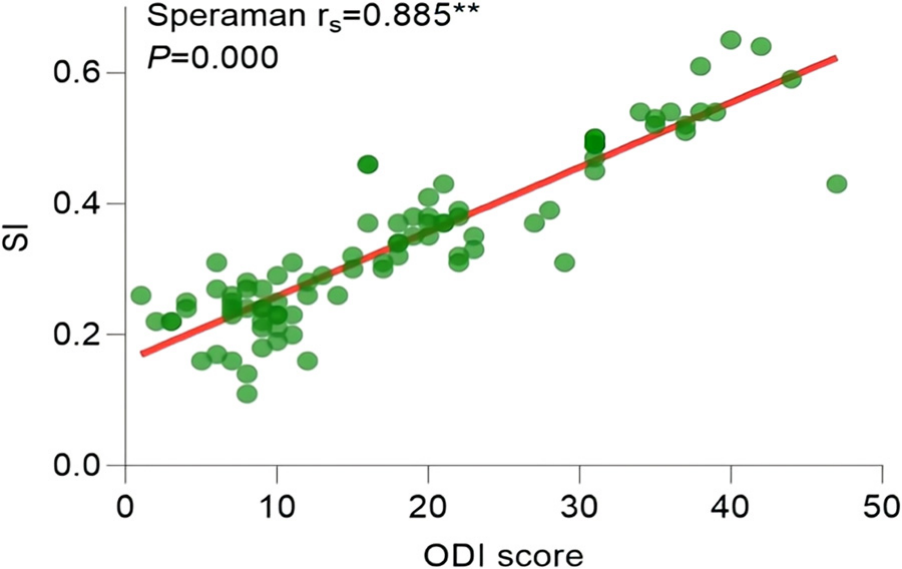
Correlation analysis between SI and ODI.

**Figure 16:**
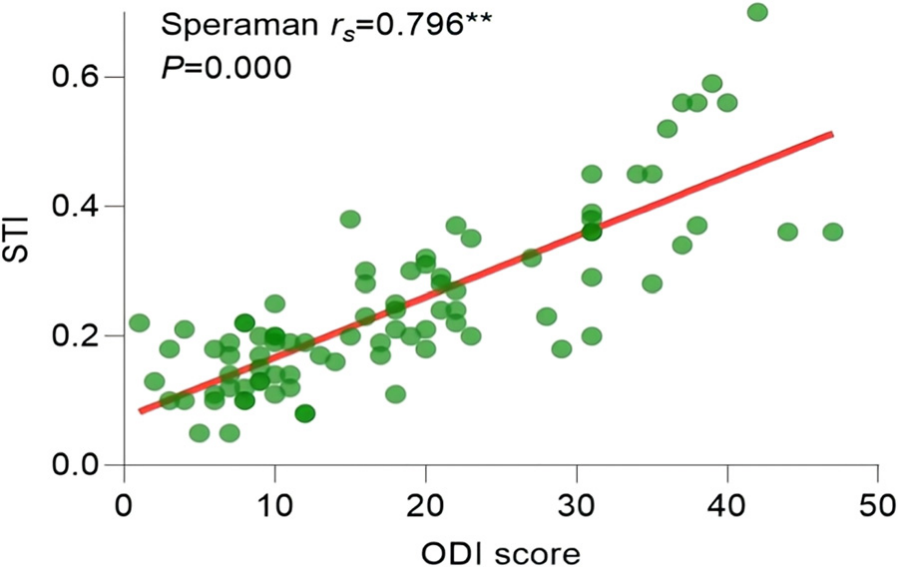
Correlation analysis between STI and ODI.

**Figure 17:**
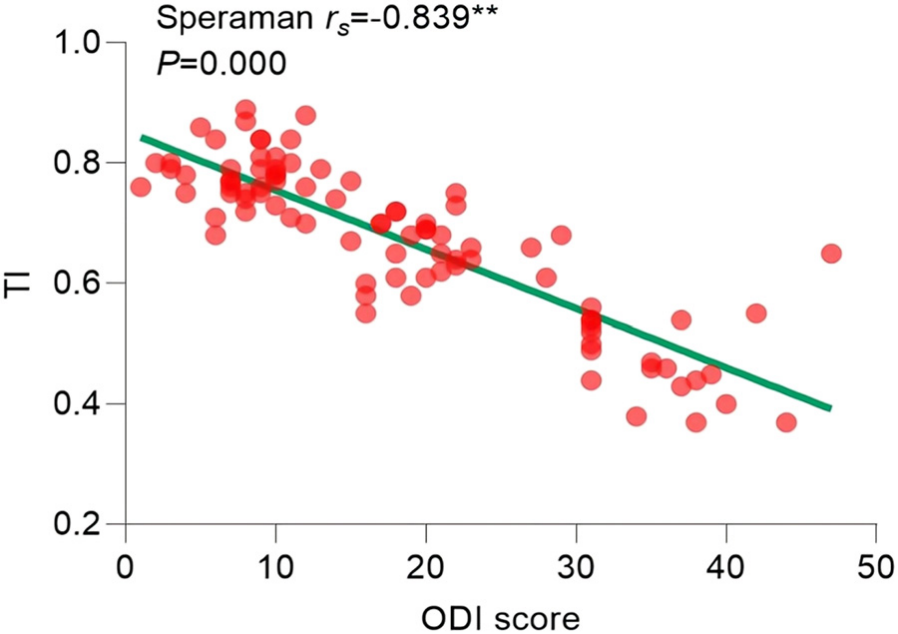
Correlation analysis between TI and ODI.

**Figure 18:**
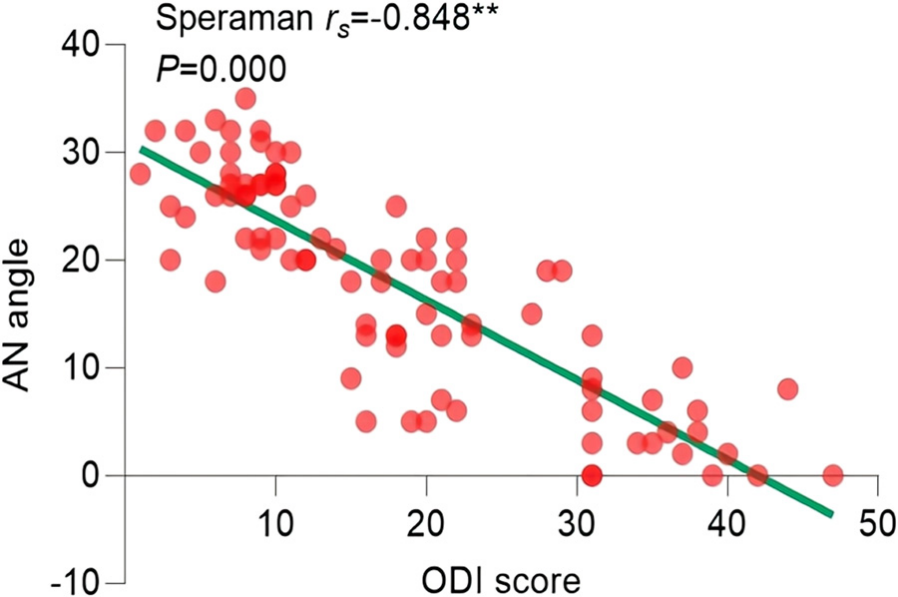
Correlation analysis between AN angle and ODI.

Since the ODI index did not follow a normal distribution (*P* > 0.05), the correlation analysis between statistically significant imaging features in pairwise comparisons and the ODI index was conducted using Spearman’s correlation. The results of the correlation analysis showed the following: the Pfirrmann classification correlation coefficient ( \[{r}_{{\mathrm{s}}}]\] = 0.674, *P* = 0.000) (Figure 12), MSU protrusion size classification correlation coefficient ( \[{r}_{{\mathrm{s}}}]\] = 0.688, *P* = 0.000) (Figure 13), MSU protrusion location classification correlation coefficient ( \[{r}_{{\mathrm{s}}}]\] = 0.744, *P* = 0.000) (Figure 14), SI correlation coefficient ( \[{r}_{{\mathrm{s}}}]\] = 0.885, *P* = 0.000) (Figure 15), and STI correlation coefficient ( \[{r}_{{\mathrm{s}}}]\] = 0.796, *P* = 0.000) (Figure 16) were positively correlated with the degree of pain. TI correlation coefficient ( \[{r}_{{\mathrm{s}}}]\] =−0.839, *P* = 0.000) (Figure 17) and AN Angle correlation coefficient ( \[{r}_{{\mathrm{s}}}]\] = −0.848, *P* = 0.000) (Figure 18) were negatively correlated with the degree of pain. All imaging features showed significant correlations with the degree of pain, as shown in Table 4.

**Table 4:** Spearman rank correlation analysis

	Pfirrmann classification	MSU protrusion size classification	MSU protrusion location classification	SI	STI	TI	AN angle (°)
Correlation coefficient	0.674**	0.688**	0.744**	0.885**	0.796**	−0.839**	−0.848**
*P*	0.000	0.000	0.000	0.000	0.000	0.000	0.000

** At the 0.01 level (two-way), correlation is significant.

**Figure 19:**
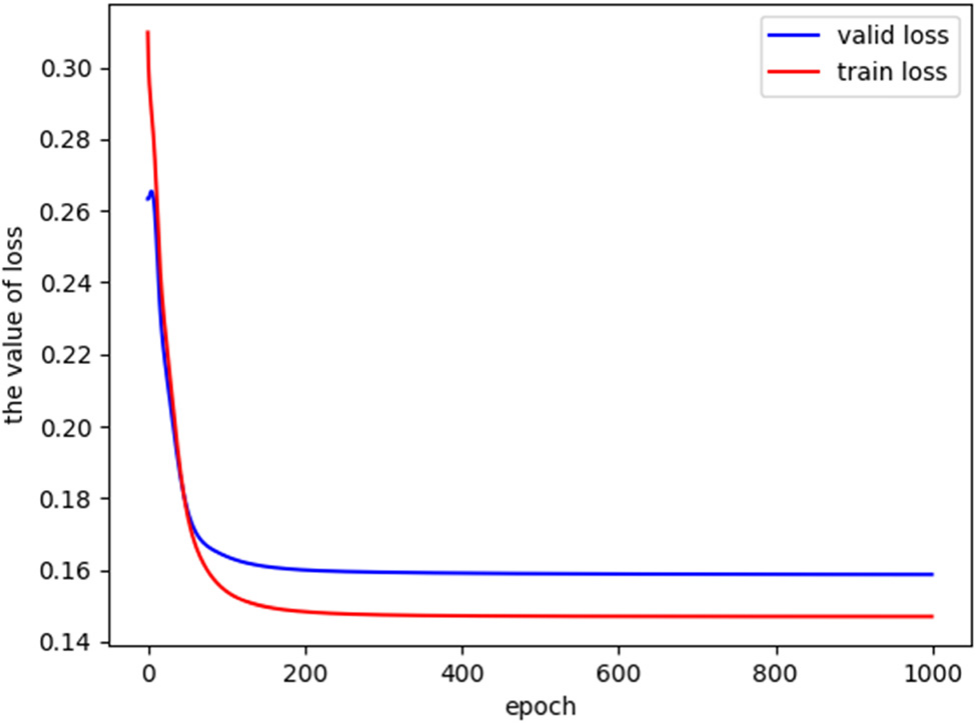
BP neural network training loss function value drop plot.

**Figure 20:**
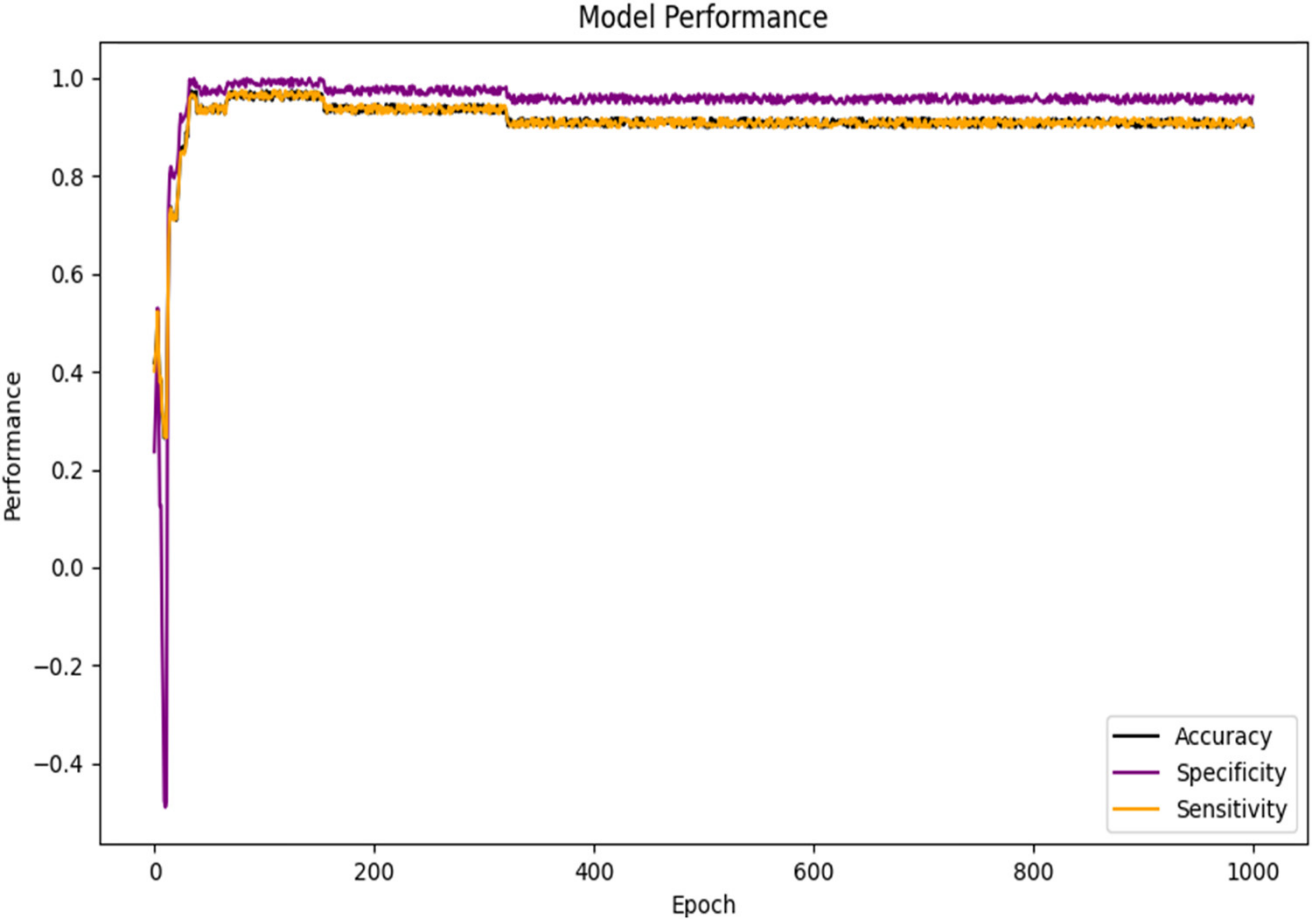
Specificity, sensitivity, and accuracy of BP neural network model.

After training, the value of loss function decreased significantly (Figure 19). In addition, after 1,000 rounds of testing, the visualization results of model specificity (95 ± 2%, mean value 95.63%), sensitivity (91 ± 2%, mean value 91.08%), and accuracy (91 ± 2%, mean value 91.03%) are shown in Figure 20.

The loss function of BP neural network model decreased, the model converged, gradually tended to be stable, and the recognition accuracy increased.

The higher the specificity, sensitivity, and accuracy, the better the performance of the model.

**Figure 21:**
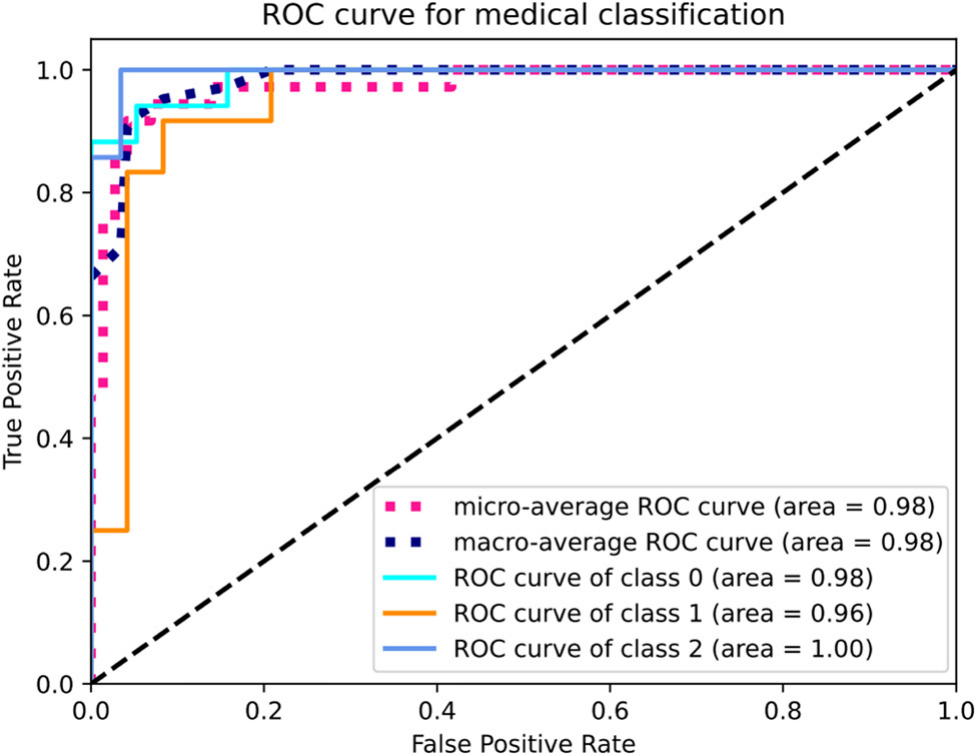
Multi-class ROC curve.

Under the condition of multi-classification, we evaluated the performance of the model, drew the receiver operating characteristic (ROC) curve of the model, and solved the area AUC under the ROC curve, as shown in Figure 21. The area of ROC curve of class 0 for predicting mild symptom group was 0.98 (*P* = 0.03). The area of ROC curve of class 1 for predicting moderate symptom group was 0.96 (*P* = 0.05). The area of ROC curve of class 2 for predicting severe symptom group was 1.00 (*P* = 0.01). The AUC under the ROC curve was ideal, which proved the success of the model training. The processing efficiency of the model network in this study showed that the average reasoning time of a single case was 50 ms.

ROC curve of class 1 was the ROC curve drawn with the first class as the positive class. Macro-average ROC curve and micro-average ROC curve were the ROC curves of macro average and micro average, respectively.

## 4 Discussion

In this study, the following imaging features were selected to evaluate the correlation between degenerative LDH and pain: Pfirrmann classification for assessing disc degeneration level, Modic classification for evaluating cartilage endplate signal changes, ligamentum flavum hypertrophy degree, and intraspinal occupation indicators (MSU regional localization, SI, STI, TI, and AN angle). These imaging features were chosen to eliminate the adverse influence of intervertebral canal variations among different patients.

In this study, the improved Pfirrmann grading system was used for IDD. The higher the grade, the more serious the degree of intervertebral disc degeneration. The results of this study demonstrated a significant correlation between disc degeneration and LDH pain symptoms, suggesting that the degree of disc degeneration generally reflects the distribution of LDH severity. The conclusion drawn is that the more severe the lumbar disc degeneration, the more significant the LDH pain symptoms. This finding is consistent with the study conducted by Faur et al. [33] on the correlation between lumbar disc degeneration and multifidus muscle (LMM) fat atrophy in patients with lower back pain, which found a higher rate of LMM atrophy in the L5/S1 segment and its correlation with disc degeneration. It is also in line with the research conducted by Foizer et al. [34], who discovered that chronic idiopathic lower back pain patients often have moderate to severe lumbar disc degeneration.

If the pressure on the endplate exceeds its limit, microfractures can occur in the subchondral bone trabeculae, leading to local changes in the spine and resulting in Modic changes. In 1987, de Roos et al. first reported changes in vertebral bone marrow signal near the endplate region in MRI images of patients with lumbar disc degeneration [35]. In 1988, Modic systematically proposed the classification of degenerative lesions in the vertebral endplate and subchondral bone signal changes on MRI, along with histological changes [24,25,26]. These changes include bone marrow edema, fatty degeneration, or sclerosis [36]. Modic changes can reflect the severity of clinical lumbar disc degeneration [37], and Kokkonen et al. also confirmed the association between endplate degeneration and lumbar disc degeneration, suggesting that endplate degeneration may be a result rather than a cause of disc degeneration [38]. In our study, out of the 90 cases, endplate changes were not observed in 60 cases. The limited number of positive results did not yield statistically significant differences in Modic classification between pairwise comparisons, thus the correlation between endplate degeneration and the severity of LDH symptoms could not be confirmed. This finding contradicts the results of studies suggesting a high diagnostic value of Modic changes for discogenic LBP [39]. However, a systematic review also found no association between Modic classification and LBP-related outcomes [40].

The ligamentum flavum is distributed between adjacent vertebral laminae and is rich in elastic fibers, making it tough and thick. Due to its close proximity to the dura mater, thickening of the ligamentum flavum can cause compression and irritation of the dura mater. In addition, most patients with ligamentum flavum hypertrophy will also experience symptoms of nerve root canal stenosis. Due to its high content of elastic fibers, the ligamentum flavum appears as a “V” shape on axial MRI images, allowing clear visualization of its thickening and deformation. This enables an accurate assessment of the degree of compression on the dura mater and the extent of spinal canal narrowing. However, in this study, statistical analysis revealed that there was no significant statistical difference observed in the thickness of the ligamentum flavum within the LDH pain group. Therefore, there is no evidence to support any correlation between ligamentum flavum hypertrophy and LDH symptoms.

MSU regional localization is an assessment criterion for evaluating the size and location of lumbar disc protrusions within and outside the spinal canal. The size of the protruded nucleus pulposus is categorized into three levels: 1, 2, and 3. The location of the protrusion is classified into four regions: A, B, AB, and C. From an anatomical perspective, regions B and C appear narrower than region A. Therefore, when the protruded disc is located in regions B or C, it usually indicates more severe compression of the spinal nerves. In this study, both the MSU protrusion size classification and the MSU protrusion location classification showed statistical significance among the three groups. Furthermore, both the MSU protrusion size classification and the MSU protrusion location classification were significantly correlated with pain symptom scores, indicating that the size and location of the protrusion are important imaging features for evaluating LDH symptoms.

The AN angle serves as an indicator of the proximity of the protruding nucleus pulposus to the intervertebral space and the level of compression exerted on the nerve root. The results of this study showed a significant correlation between the AN angle and the degree of pain, which is consistent with the findings of Kobayashi et al. [41]. Their findings indicated that nerve root compression led to reduced blood flow and neuronal cell count, consequently impacting neurotransmitter metabolism and resulting in impaired nerve function.

The study results demonstrated significant correlations between the following indicators of intraspinal occupancy: MSU regional localization (MSU protrusion size classification and MSU protrusion location classification), SI, STI, TI, AN angle, and LDH pain. These findings are consistent with Thapa et al.’s study [42], which analyzed the correlation between clinical characteristics and MRI-visible abnormal imaging features (such as disc protrusion type, location, nerve root, and intervertebral foramen damage) in LDH patients. Thapa found a good correlation between disc protrusion with significant compression of the nerve root and clinical features, supporting the theory of mechanical compression. The theory of mechanical compression is currently the most widely accepted view due to its directness. According to this theory, the protruded nucleus pulposus of the intervertebral disc directly or indirectly compresses the nerve root and/or the dura mater, affecting the normal blood supply to the nerve root and inducing symptoms of nerve root irritation. Mixter and Barr [43] first reported sciatic nerve pain associated with LDH, suggesting that it is caused by mechanical injury to the nerve root, with the main factors being indirect compression of the spinal nerve root and the tension effect on the nerve root. The symptoms are related to changes in the position of the protruded disc tissue, the transmission of pain information within the nervous system, and the interaction between the nucleus pulposus, dura mater, or nerve root. In addition, it may also be related to local microcirculation and inflammatory changes caused by protruded nucleus pulposus, as well as osteophyte degeneration and changes in lumbar stress posture. However, clinical studies have found that many asymptomatic individuals still show compressive changes in LDH imaging [44,45,46]. Whether the theory of mechanical compression alone can cause pain requires further investigation.

During the degenerative process of lumbar intervertebral discs, there is an increase in the number of senescent cells. It induces the upregulation of pro-inflammatory cytokines, chemokines, and tissue-damaging proteases, including tumor necrosis factor-alpha (TNF-α) and interleukin-1 alpha (IL-1α). This can induce local inflammation, leading to neurological signs such as radicular pain and exacerbating pain in patients with LDH [47]. Pain signals originating from the intervertebral disc can be transmitted through adjacent structures via peripheral nerve fibers. The interaction between nerve fibers and inflammatory mediators in the nucleus pulposus can trigger lower back pain. However, this study focuses primarily on the analysis of LDH imaging features and cannot confirm whether the pain associated with LDH is related to the chemical radiculitis theory.

In recent years, the development of machine learning has greatly enhanced the ability to handle complex and large amounts of information, particularly in the field of medical diagnosis. Numerous researchers have utilized machine learning techniques for in-depth automated grading and predictive diagnosis of diseases. For instance, Jamaludin trained a convolutional neural network to automatically grade lumbar intervertebral discs and vertebral bodies [48]. Abdel-Zaher and Eldeib designed a neural network, DBN-NN, for automatic diagnosis of breast cancer, achieving an overall accuracy of 99.68%, sensitivity of 100%, and specificity of 99.47% [49]. Kleesiek et al. conducted work on the classification of lung nodules, achieving an accuracy of 75.01% and sensitivity of 83.35%. In a 10-fold cross-validation, the false positive rate per patient was 0.39 [50]. Wei and Yang discovered that a back propagation artificial neural network model demonstrated higher predictive accuracy for non-invasive diagnosis of digestive system diseases compared to linear regression [51].

The BP neural network is a multi-layer feedforward neural network structure developed by McClelland and Rumelhart for supervised learning [52]. It uses a variety of training samples to modify the weights in order to minimize the error value. The error function is obtained through a recursive process of BP starting from the output layer. Therefore, it is based on the backpropagation algorithm and is also known as an error BP neural network.

The design of a multi-layer neural network enables the BP neural network to learn more feature information from the input, thus completing more complex classification tasks. It is suitable for simulating the approximate relationship between input and output in computational modeling. The network operation involves two steps: forward propagation and backward learning. During forward propagation, the input data passes through the hidden layers layer by layer from the input layer. During network training, optimization is performed in the direction that reduces the value of the loss function. the value of the loss function becomes smaller and smaller. In the reverse learning process, numerical adjustments are made from the output layer to the input layer to achieve the desired output of the network. With the deepening of the learning process of BP neural network, its performance in complex model fitting and distribution approximation is obviously superior to the traditional statistical methods. [53]. The BP neural network transforms linear data into nonlinear data through activation functions, making it capable of handling linearly inseparable problems, which aligns with the characteristics of the data in this study.

Prior literature research revealed lack of studies applying the BP neural network model to LDH research. Hence, developing an LDH pain prediction model based on the BP neural network holds significant importance for LDH diagnosis and pathogenesis investigation. In this study, we successfully developed and validated a BP neural network for automated LDH pain grading, which displayed impressive performance in pain classification. The study results indicated the BP neural network model’s convergence and approaching stability, as evidenced by the decreasing loss and increasing recognition accuracy. The BP neural network training model results showed the specificity (95 ± 2%, mean value: 95.63%), sensitivity (91 ± 2%, mean value: 91.08%), and accuracy (91 ± 2%, mean value: 91.03%) of the model. The area under the ROC curve for predicting pain type 0 (mild), type 1 (moderate), and type 2 (severe) were 0.98, 0.96, and 1.00, respectively. Compared to similar studies, the results of this study are at a relatively high level. The macro-average ROC curve and micro-average ROC curve are average indicators for multi-classification problems. The high values of both micro-average and macro-average (both above 95%) indicate good classification performance of the model in this study.

In this study, we meticulously weighed the pros and cons of the BP neural network and strategically employed suitable techniques to address its limitations. Consequently, the BP neural network’s performance reached an impressive level. Despite the small data size, the utilization of oversampling techniques, and *k*-fold cross-validation effectively mitigated overfitting concerns. This successful approach validated the BP model’s predictive classification capability for assessing the degree of LDH pain.

## References

[1] Andersson GB. Epidemiology of low back pain. Acta Orthop Scand Suppl. 1998;281:28–31. 10.1080/17453674.1998.11744790 9771538

[2] Meucci RD, Fassa AG, Faria NM. Prevalence of chronic low back pain: systematic review. Rev Saude Publica. 2015;49:1. 10.1590/S0034-8910.2015049005874 PMC4603263 26487293

[3] Hartvigsen J, Hancock MJ, Kongsted A, Louw Q, Ferreira ML, Genevay S, et al. What low back pain is and why we need to pay attention. Lancet. 2018;391(10137):2356–67. 10.1016/S0140-6736(18)30480-X 29573870

[4] Airaksinen O, Brox JI, Cedraschi C, Hildebrandt J, Klaber-Moffett J, Kovacs F, et al. Chapter 4. European guidelines for the management of chronic nonspecific low back pain. Eur Spine J. 2006;15(Suppl 2):S192–300. 10.1007/s00586-006-1072-1 PMC3454542 16550448

[5] Murray CJ, Vos T, Lozano R, Naghavi M, Flaxman AD, Michaud C, et al. Disability-adjusted life years (DALYs) for 291 diseases and injuries in 21 regions, 1990-2010: a systematic analysis for the Global Burden of Disease Study 2010. Lancet. 2012;380(9859):2197–223. 10.1016/S0140-6736(12)61689-4 23245608

[6] Weber H. The natural history of disc herniation and the influence of intervention. Spine (Phila Pa 1976). 1994;19(19):2234–8 discussion 2233. 10.1097/00007632-199410000-00022 7809761

[7] Kreiner DS, Hwang SW, Easa JE, Resnick DK, Baisden JL, Bess S, et al. An evidence-based clinical guideline for the diagnosis and treatment of lumbar disc herniation with radiculopathy. Spine J. 2014;14(1):180–91. 10.1016/j.spinee.2013.08.003 24239490

[8] Liu C, Xue J, Liu J, Ma G, Moro A, Liang T, et al. Is there a correlation between upper lumbar disc herniation and multifidus muscle degeneration? A retrospective study of MRI morphology. BMC Musculoskelet Disord. 2021;22(1):92. 10.1186/s12891-021-03970-x PMC7814711 33468108

[9] Chen D, He S. Value of lumbar MRI parameters in the evaluation of postoperative curative effect on patients with lumbar disc herniation and analysis of risk factors. Evid Based Complement Altern Med. 2021;2021:4514704. 10.1155/2021/4514704 PMC8553447 34721632

[10] Yang S, Liu Y, Bao Z, Zou J, Yang H. Comparison of adjacent segment degeneration after nonrigid fixation system and posterior lumbar interbody fusion for single-level lumbar disc herniation: A new method of MRI analysis of lumbar nucleus pulposus volume. J Invest Surg. 2018;31(4):307–12. 10.1080/08941939.2017.1325542 28525292

[11] Porchet F, Wietlisbach V, Burnand B, Daeppen K, Villemure JG, Vader JP. Relationship between severity of lumbar disc disease and disability scores in sciatica patients. Neurosurgery. 2002;50(6):1253–9 discussion 1259–60. 10.1097/00006123-200206000-00014 12015843

[12] Vanharanta H, Floyd T, Ohnmeiss DD, Hochschuler SH, Guyer RD. The relationship of facet tropism to degenerative disc disease. Spine (Phila Pa 1976). 1993;18(8):1000–5. 10.1097/00007632-199306150-00008 8367766

[13] Sasaki T, Yoshimura N, Hashizume H, Yamada H, Oka H, Matsudaira K, et al. MRI-defined paraspinal muscle morphology in Japanese population: The Wakayama Spine Study. PLoS One. 2017;12(11):e0187765. 10.1371/journal.pone.0187765 PMC5678698 29117256

[14] Dunsmuir RA, Nisar S, Cruickshank JA, Loughenbury PR. No correlation identified between the proportional size of a prolapsed intravertebral disc with disability or leg pain. Bone Jt J. 2022;104-B(6):715–20. 10.1302/0301-620X.104B6.BJJ-2021-1725.R2 35638217

[15] Ranger TA, Teichtahl AJ, Cicuttini FM, Wang Y, Wluka AE, OʼSullivan R, et al. Shorter lumbar paraspinal fascia is associated with high intensity low back pain and disability. Spine (Phila Pa 1976). 2016;41(8):E489–93. 10.1097/BRS.0000000000001276 27064338

[16] Ley C, Martin RK, Pareek A, Groll A, Seil R, Tischer T. Machine learning and conventional statistics: making sense of the differences. Knee Surg Sports Traumatol Arthrosc. 2022;30(3):753–7. 10.1007/s00167-022-06896-6 35106604

[17] Eriksson S, Waldenberg C, Torén L, Grimby-Ekman A, Brisby H, Hebelka H, et al. Texture analysis of magnetic resonance images enables phenotyping of potentially painful annular fissures. Spine (Phila Pa 1976). 2022;47(5):430–7. 10.1097/BRS.0000000000004160 34265808

[18] Su ZH, Liu J, Yang MS, Chen ZY, You K, Shen J, et al. Automatic grading of disc herniation, central canal stenosis and nerve roots compression in lumbar magnetic resonance image diagnosis. Front Endocrinol (Lausanne). 2022;13:890371. 10.3389/fendo.2022.890371 PMC9207332 35733770

[19] Abdollah V, Parent EC, Dolatabadi S, Marr E, Croutze R, Wachowicz K, et al. Texture analysis in the classification of T2-weighted magnetic resonance images in persons with and without low back pain. J Orthop Res. 2021;39(10):2187–96. 10.1002/jor.24930 33247597

[20] Hopkins BS, Weber KA, Kesavabhotla K, Paliwal M, Cantrell DR, Smith ZA. Machine learning for the prediction of cervical spondylotic myelopathy: A post hoc pilot study of 28 participants. World Neurosurg. 2019;127:e436–42. 10.1016/j.wneu.2019.03.165 PMC6610711 30922901

[21] Yushkevich PA, Piven J, Hazlett HC, Smith RG, Ho S, Gee JC, et al. User-guided 3D active contour segmentation of anatomical structures: significantly improved efficiency and reliability. Neuroimage. 2006;31(3):1116–28. 10.1016/j.neuroimage.2006.01.015 16545965

[22] Pfirrmann CW, Metzdorf A, Zanetti M, Hodler J, Boos N. Magnetic resonance classification of lumbar intervertebral disc degeneration. Spine (Phila Pa 1976). 2001;26(17):1873–8. 10.1097/00007632-200109010-00011 11568697

[23] Griffith JF, Wang YX, Antonio GE, Choi KC, Yu A, Ahuja AT, et al. Modified Pfirrmann classification system for lumbar intervertebral disc degeneration. Spine (Phila Pa 1976). 2007;32(24):E708–12. 10.1097/BRS.0b013e31815a59a0 18007231

[24] Modic MT, Masaryk TJ, Ross JS, Carter JR. Imaging of degenerative disk disease. Radiology. 1988;168(1):177–86. 10.1148/radiology.168.1.3289089 3289089

[25] Modic MT, Steinberg PM, Ross JS, Masaryk TJ, Carter JR. Degenerative disk disease: assessment of changes in vertebral body marrow with MR imaging. Radiology. 1988;166(1 Pt 1):193–9. 10.1148/radiology.166.1.3336678 3336678

[26] Fayad F, Lefevre-Colau MM, Drapé JL, Feydy A, Chemla N, Quintéro N, et al. Reliability of a modified Modic classification of bone marrow changes in lumbar spine MRI. Jt Bone Spine. 2009;76(3):286–9. 10.1016/j.jbspin.2008.09.012 19119042

[27] Mysliwiec LW, Cholewicki J, Winkelpleck MD, Eis GP. MSU classification for herniated lumbar discs on MRI: toward developing objective criteria for surgical selection. Eur Spine J. 2010;19(7):1087–93. 10.1007/s00586-009-1274-4 PMC2900017 20084410

[28] Thelander U, Fagerlund M, Friberg S, Larsson S. Describing the size of lumbar disc herniations using computed tomography. A comparison of different size index calculations and their relation to sciatica. Spine (Phila Pa 1976). 1994;19(17):1979–84. 10.1097/00007632-199409000-00020 7997933

[29] Guo W, Zhao P, Zhou W, Wei J, Li XD, Zhou H, et al. Correlation studies between MRI and the symptom scores of patients with LDH before and after manipulative therapy. Zhongguo Gu Shang. 2010;23(1):17–9. 20191957

[30] Wilmink JT. CT morphology of intrathecal lumbosacral nerve-root compression. AJNR Am J Neuroradiol. 1989;10(2):233–48. PMC8331366 2494846

[31] Sakamaki T, Sairyo K, Sakai T, Tamura T, Okada Y, Mikami H. Measurements of ligamentum flavum thickening at lumbar spine using MRI. Arch Orthop Trauma Surg. 2009;129(10):1415–9. 10.1007/s00402-009-0849-1 19280205

[32] Fairbank JC, Pynsent PB. The Oswestry disability index. Spine (Phila Pa 1976). 2000;25(22):2940–52 discussion 2952. 10.1097/00007632-200011150-00017 11074683

[33] Faur C, Patrascu JM, Haragus H, Anglitoiu B. Correlation between multifidus fatty atrophy and lumbar disc degeneration in low back Pain. BMC Musculoskelet Disord. 2019;20(1):414. 10.1186/s12891-019-2786-7 PMC6729014 31488112

[34] Foizer GA, Paiva VC, Nascimento RDD, Gorios C, Cliquet Júnior A, Miranda JB. Is there any association between the severity of disc degeneration and low back pain? Rev Bras Ortop (Sao Paulo). 2021;57(2):334–40. 10.1055/s-0041-1735831 PMC9142238 35652022

[35] de Roos A, Kressel H, Spritzer C, Dalinka M. MR imaging of marrow changes adjacent to end plates in degenerative lumbar disk disease. AJR Am J Roentgenol. 1987;149(3):531–4. 10.2214/ajr.149.3.531 3497539

[36] Sokov EL, Kornilova LE, Nesterov AI. Poiasnichnaia bol’ i izmeneniia pozvonkov po tipu Modik [Low back pain and Modic changes]. Zh Nevrol Psikhiatr Im S S Korsakova. 2017;117(6):99–105. 10.17116/jnevro20171176199-105 28745679

[37] Xiao L, Ni C, Shi J, Wang Z, Wang S, Zhang J, et al. Analysis of correlation between vertebral endplate change and lumbar disc degeneration. Med Sci Monit. 2017;23:4932–8. 10.12659/MSM.904315 PMC5655151 29032381

[38] Kokkonen SM, Kurunlahti M, Tervonen O, Ilkko E, Vanharanta H. Endplate degeneration observed on magnetic resonance imaging of the lumbar spine: correlation with pain provocation and disc changes observed on computed tomography diskography. Spine (Phila Pa 1976). 2002;27(20):2274–8. 10.1097/00007632-200210150-00017 12394906

[39] Song J, Wang HL, Ma XS, Xia XL, Lu FZ, Zheng CJ, et al. The value of radiographic indexes in the diagnosis of discogenic low back pain: a retrospective analysis of imaging results. Oncotarget. 2017;8(36):60558–67. 10.18632/oncotarget.18652 PMC5601161 28947993

[40] Herlin C, Kjaer P, Espeland A, Skouen JS, Leboeuf-Yde C, Karppinen J, et al. Modic changes-Their associations with low back pain and activity limitation: A systematic literature review and meta-analysis. PLoS One. 2018;13(8):e0200677. 10.1371/journal.pone.0200677 PMC6070210 30067777

[41] Kobayashi S, Kokubo Y, Uchida K, Yayama T, Takeno K, Negoro K, et al. Effect of lumbar nerve root compression on primary sensory neurons and their central branches: changes in the nociceptive neuropeptides substance P and somatostatin. Spine (Phila Pa 1976). 2005;30(3):276–82. 10.1097/01.brs.0000152377.72468.f4 15682006

[42] Thapa SS, Lakhey RB, Sharma P, Pokhrel RK. Correlation between clinical features and magnetic resonance imaging findings in lumbar disc prolapse. J Nepal Health Res Counc. 2016;14(33):85–8. 27885288

[43] Mixter WJ, Barr JS. Rupture of the intervertebral disc with involvement of the spinal canal. N Englan J Med. 1934;211:210–5.

[44] Jensen MC, Brant-Zawadzki MN, Obuchowski N, Modic MT, Malkasian D, Ross JS. Magnetic resonance imaging of the lumbar spine in people without back Pain. N Engl J Med. 1994;331(2):69–73. 10.1056/NEJM199407143310201 8208267

[45] Boos N, Rieder R, Schade V, Spratt KF, Semmer N, Aebi M. Volvo award in clinical sciences. The diagnostic accuracy of magnetic resonance imaging, work perception, and psychosocial factors in identifying symptomatic disc herniations. Spine (Phila Pa 1976). 1995;20(24):2613–25. 10.1097/00007632-199512150-00002 8747239

[46] Boden SD, Davis DO, Dina TS, Patronas NJ, Wiesel SW. Abnormal magnetic-resonance scans of the lumbar spine in asymptomatic subjects. A prospective investigation. J Bone Jt Surg Am. 1990;72(3):403–8. 2312537

[47] Urits I, Burshtein A, Sharma M, Testa L, Gold PA, Orhurhu V, et al. Low back pain, a comprehensive review: pathophysiology, diagnosis, and treatment. Curr Pain Headache Rep. 2019;23(3):23. 10.1007/s11916-019-0757-1 30854609

[48] Jamaludin A, Lootus M, Kadir T, Zisserman A, Urban J, Battié MC, et al. ISSLS prize in bioengineering science 2017: Automation of reading of radiological features from magnetic resonance images (MRIs) of the lumbar spine without human intervention is comparable with an expert radiologist. Eur Spine J. 2017;26(5):1374–83. 10.1007/s00586-017-4956-3 28168339

[49] Abdel-Zaher AM, Eldeib AM. Breast cancer classification using deep belief networks. Expert Syst Appl. 2016;46:139–44.

[50] Kleesiek J, Urban G, Hubert A, Schwarz D, Maier-Hein K, Bendszus M, et al. Deep MRI brain extraction: A 3D convolutional neural network for skull stripping. Neuroimage. 2016;129:460–9. 10.1016/j.neuroimage.2016.01.024 26808333

[51] Wei W, Yang X. Comparison of diagnosis accuracy between a backpropagation artificial neural network model and linear regression in digestive disease patients: an empirical research. Comput Math Methods Med. 2021;2021:6662779. 10.1155/2021/6662779 PMC7937476 33727951

[52] McClelland JL, Rumelhart DE. Distributed memory and the representation of general and specific information. J Exp Psychol Gen. 1985;114(2):159–97. 10.1037//0096-3445.114.2.159 3159828

[53] Lyu J, Zhang J. BP neural network prediction model for suicide attempt among Chinese rural residents. J Affect Disord. 2019;246:465–73. 10.1016/j.jad.2018.12.111 PMC6430644 30599370

